# Molecular insights into the mechanisms of susceptibility of *Labeo rohita* against oomycete *Aphanomyces invadans*

**DOI:** 10.1038/s41598-020-76278-w

**Published:** 2020-11-11

**Authors:** P. K. Pradhan, Dev Kumar Verma, Luca Peruzza, Shubham Gupta, Syed Assim Haq, Sergei V. Shubin, Kenton L. Morgan, Franziska Trusch, Vindhya Mohindra, Chris Hauton, Pieter van West, Neeraj Sood

**Affiliations:** 1 ICAR-National Bureau of Fish Genetic Resources, Canal Ring Road, P.O. Dilkusha, Lucknow, Uttar Pradesh 226 002 India; 2 School of Ocean and Earth Science, University of Southampton, Waterfront Campus, European Way, Southampton, SO14 3ZH United Kingdom; 3 College of Science, Swansea University, Singleton Park, Swansea, SA2 8PP Wales United Kingdom; 4 The Institute of Veterinary Science, University of Liverpool, Leahurst Campus, Neston, Liverpool, CH64 7TE United Kingdom; 5 International Centre for Aquaculture Research and Development, Institute of Medical Sciences, University of Aberdeen, Foresterhill, Aberdeen, AB25 2ZD Scotland United Kingdom; 6 Present Address: Department of Comparative Biomedicine and Food Science, University of Padova, Viale dell’Università 16, 35020 Legnaro (PD), Italy; 7 Present Address: University of Dundee, School of Life Sciences, Department of Plant Sciences (@ James Hutton Institute), Invergowrie, Dundee, DD2 5DA Scotland United Kingdom

**Keywords:** Immunology, Diseases

## Abstract

*Aphanomyces invadans*, the causative agent of epizootic ulcerative syndrome, is one of the most destructive pathogens of freshwater fishes. To date, the disease has been reported from over 160 fish species in 20 countries and notably, this is the first non-salmonid disease that has resulted in major impacts globally. In particular, Indian major carps (IMCs) are highly susceptible to this disease. To increase our knowledge particularly with regards to host immune response against *A. invadans* infection in a susceptible host, the gene expression profile in head kidney of *A. invadans*-infected and control rohu, *Labeo rohita* was investigated using RNA sequencing. Time course analysis of RNA-Seq data revealed 5608 differentially expressed genes, involved among others in Antigen processing and presentation, Leukocyte transendothelial migration, IL-17 signaling, Chemokine signaling, C-type lectin receptor signaling and Toll-like receptor signaling pathways. In the affected pathways, a number of immune genes were found to be downregulated, suggesting an immune evasion strategy of *A. invadans* in establishing the infection. The information generated in this study offers first systematic mechanistic understanding of the host–pathogen interaction that might underpin the development of new management strategies for this economically devastating fish-pathogenic oomycete *A. invadans*.

## Introduction

Infection with *Aphanomyces invadans* is a serious disease of freshwater fishes that is characterized by the presence of oomycete hyphae and necrotizing ulcerative lesions, leading to pathognomonic granulomatous response^1,2^. The disease, commonly known as epizootic ulcerative syndrome (EUS), was first reported from ayu in Japan^3^ and has subsequently been reported from 20 countries across 4 continents^1^ and the disease is spreading to newer areas^4–6^. To date, more than 160 species of fish have been reported to be susceptible to *A. invadans*^2^ and the host range is expanding^7–10^. Furthermore, it is believed that many more species are likely to be susceptible to *A. invadans* infection^2^. Importantly, due to its broad host range, ability to cause epizootics and potential socio-economic impacts, infection with *A. invadans* has been included in finfish diseases reportable to World Organization for Animal Health (OIE)^11,12^. However, despite being one of the most destructive diseases of fish worldwide, scarce information is available about the fundamental mechanisms involved in host response to *A. invadans* infection^13^.

During development of an infection, pathogens try to invade host by manipulating cellular pathways for their own survival and replication, whereas, host cells respond to the invading pathogen by altering gene expression in favor of defense mechanisms^14^. Since the interaction between host and pathogen is dynamic and varies during the course of infection, therefore, it is essential to characterize the temporal changes in gene expression^15,16^. RNA sequencing (RNA-Seq) followed by de novo transcriptome assembly enables large-scale analysis of transcriptomes to analyze gene expression^17^. Furthermore, comparison of RNA sequences from infected and uninfected samples gives insights into immune-related genes that are differentially expressed during an infection, and ultimately lead to a better understanding of molecular mechanisms involved in host immunity to a particular pathogen^18^. The head kidney is a key lymphoid organ in teleosts which plays a major role in immune response against the pathogens^19^. Therefore, herein, we have used RNA-Seq to compare the gene expression changes in head kidney of *A. invadans* infected rohu *Labeo rohita* with a non-infected control at 1, 3, 6 and 12 days post-infection (dpi). In time course analysis, 5608 genes were found to be differentially expressed, out of which, 390 genes were immune-related and associated with 21 immune pathways. Importantly, majority of key genes in Antigen processing and presentation and Toll-like receptor signaling pathway were found to be downregulated, which suggested an immune evasion strategy of *A. invadans*. The information generated in this study would provide novel insights into host defence mechanisms that are affected following *A. invadans* infection and will serve as knowledge base and platform for developing future novel disease control measures.

## Results

### Gross lesions

**Figure 1:**
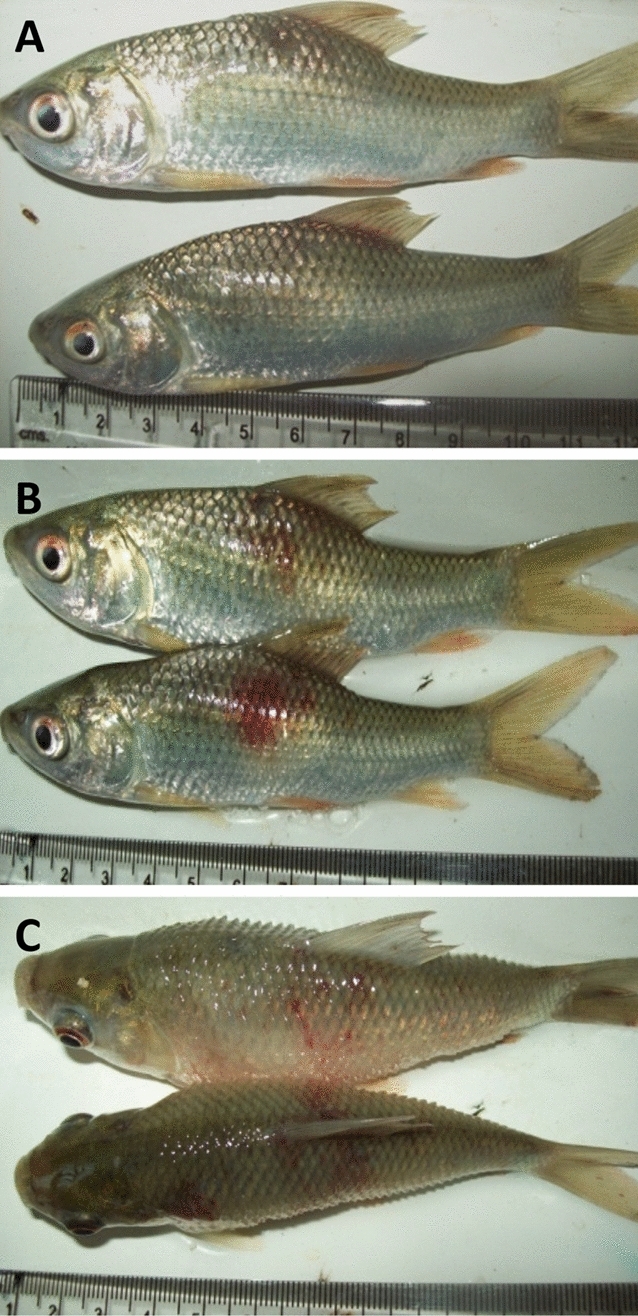
Gross lesions in rohu following experimental infection with *Aphanomyces invadans* at 3 (**A**), 6 (**B**) and 12 (**C**) days post-infection depicting early, mid and late stages of infection, respectively. Please note increase in severity of lesions with progression of infection.

Experimentally infected fish developed mild swelling at the site of injection 3 dpi followed by haemorrhagic swollen areas 6 dpi. At 12 dpi, lesions became more obvious and the haemorrhagic swelling was observed on both sides of the body (Fig. 1A–C). Gross lesions were categorised in early, mid and late stage for fish sampled at 1 and 3 dpi, 6 dpi, and 12 dpi, respectively. However, no gross lesions were observed in the control group throughout the course of the experiment.

### Transcriptome assembly

After cleaning the raw reads with Trimmomatic, a total of 457 million reads were used for de novo transcriptome assembly. These generated 1,076,144 contigs with N50 value of 282 (Table 1). The completeness and integrity of the assembled transcriptome revealed that 98.28% of the benchmarking orthologous vertebrate genes were present in the initial assembly. The sequencing statistics including number of raw, trimmed and mapped reads are listed in Suppl. Table 1. The transcriptome assembly was subsequently reduced to 58,274 contigs (N50 = 1596) after normalisation with RUVs package and removal of poorly covered contigs (Table 1).

**Table 1:** Summary of assembly statistics for the transcripts obtained from head kidney of control and *Aphanomyces invadans-*infected rohu.

Statistics	Initial assembly	CPM filter (> 1 cpm)
Assembled transcripts	1,076,144	58,274
Average length	298	822
Transcript N50	282	1596
BUSCO	98.28% Complete	–
	0.4% Fragmented	
	1.24% Missing	
Blasted transcripts	–	30,747 (52.76%)
Annotated transcripts	–	30,009 (51.49%)

Principal component analysis (PCA) of the normalized transcripts revealed that the replicates clustered together, however, there was a clear separation particularly between the control and *A. invadans*-infected samples at 1 and 12 dpi (Suppl. Figure 1).

### Functional annotation of unigenes

To identify the putative functions of transcripts, the sequences of the reduced assembly were blasted against the reference proteins available in NCBI Uniprot protein database (E-value cut off: 1E-05). A total of 30,747 unigenes (52.8%) showed significant similarity to proteins from the Uniprot database. Based on sequence similarity, 30,009 unigenes (97.6%) of these had at least one GO term assigned facilitating the functional characterisation of assembled unigenes.

### Time course (TC) differential gene expression

**Figure 2:**
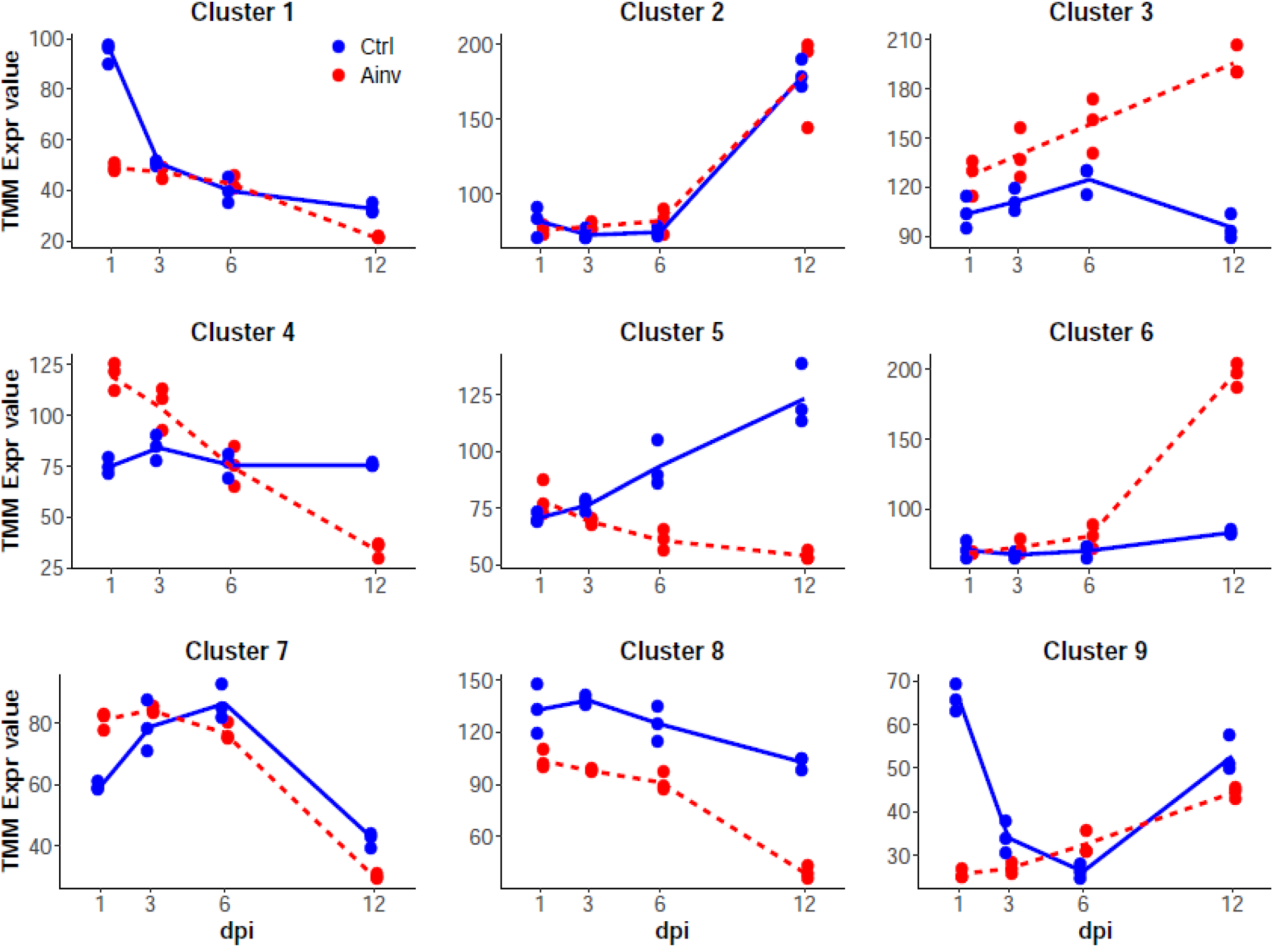
Time course analysis of clustered gene expression in *Labeo rohita* head kidney following *Aphanomyces invadans* infection. Each graph represents a cluster of genes according to their expression profile out of 5608 differentially expressed unigenes. In each graph, a dot represents the median expression level for each biological replicate (n = 24 replicates in total). For each treatment, the average expression levels at each time point are connected by a line. The Y axis represents the normalized expression levels according to the Trimmed mean of M-value (TMM) normalization algorithm. Blue dots and lines = Control, while red dots and lines = *Aphanomyces invadans*-infected rohu.

A total of 5608 unigenes with significant temporal expression changes and differences between treatments (i.e. 'Ctrl' and 'Ainv', Suppl. Tables 2 and 3) were identified in TC analysis. These unigenes were grouped in 9 different clusters of expression (Fig. 2). Unigenes in clusters 3 and 6 were upregulated, while unigenes in clusters 5 and 8 were downregulated in Ainv group. In addition, temporal changes were observed within the clusters: unigenes in cluster 4 and 7 had the highest expression level at 1 dpi whereas, in the cluster 3 and 6, maximum expression of unigenes was observed at 12 dpi. On the contrary, unigenes in clusters 5 and 8 showed maximum downregulation at 12 dpi. Furthermore, there was no difference in expression of unigenes in cluster 2 in both Ainv and Ctrl groups. Enrichment analysis of the clusters revealed an over-representation of categories involved in neutrophil degranulation, MHC class II binding protein, regulation of interferon-gamma secretion, regulation of chemokine production, lysozyme activity, positive regulation of leukocyte differentiation, Fc-gamma receptor signaling pathway involved in phagocytosis and lymphocyte chemotaxis (Suppl. Table 4).

### Cellular processes and pathways affected during *A. invadans* infection

KEGG annotation of unigenes identified by TC analysis allowed the identification of pathways that were altered in rohu head kidney during infection. Overall, differentially expressed genes (DEGs) varied between infected fish and the control group as well as between different time points of *A. invadans*-infected samples. In total, 2152 (38.37%) unigenes were assigned to different KEGG pathways. The most abundant categories included organismal systems (537 unigenes, 9.57%), cellular processes (436 unigenes, 7.77%), environmental information processing (426 unigenes, 7.59%), metabolism (389 unigenes, 6.93%) and genetic information processing (364 unigenes, 6.49%) (Suppl. Figure 2).

### Genes and pathways related to the immune system

**Figure 3:**
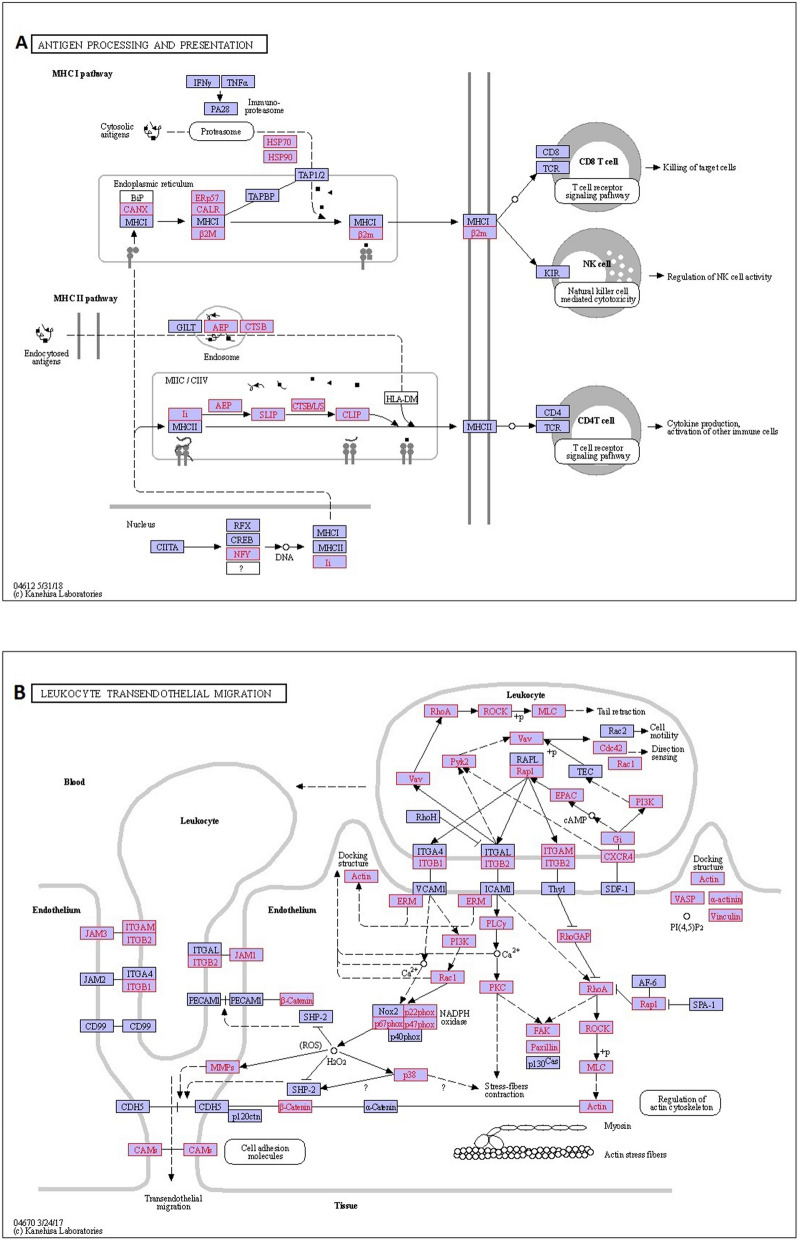
Differentially expressed genes (DEGs) involved in; (**A**) Antigen processing and presentation, and (**B**) Leukocyte transendothelial migration pathways (images reproduced with permission from KEGG)^20^. The genes in the red box represent DEGs between the *A. invadans*-infected and control *Labeo rohita* at different time points.

The main focus of this study was the potential interplay between host immunity and *A. invadans.* In the organismal systems category, the majority of DEGs (390, equivalent to 72.62% of DEGs) showed homology to immune-related pathways and covered the 21 immune pathways which included among others Antigen processing and presentation, Leukocyte transendothelial migration, IL-17 signaling, Chemokine signaling, C-type lectin receptor signaling and Toll-like receptor signaling pathways (Suppl. Table 5). The DEGs in selected immune pathways i.e. Antigen processing and presentation, and Leukocyte transendothelial migration in infected rohu are depicted in Fig. 3A,B. The contig names matching each gene and their expression values are shown in Suppl. Table 6.

**Figure 4:**
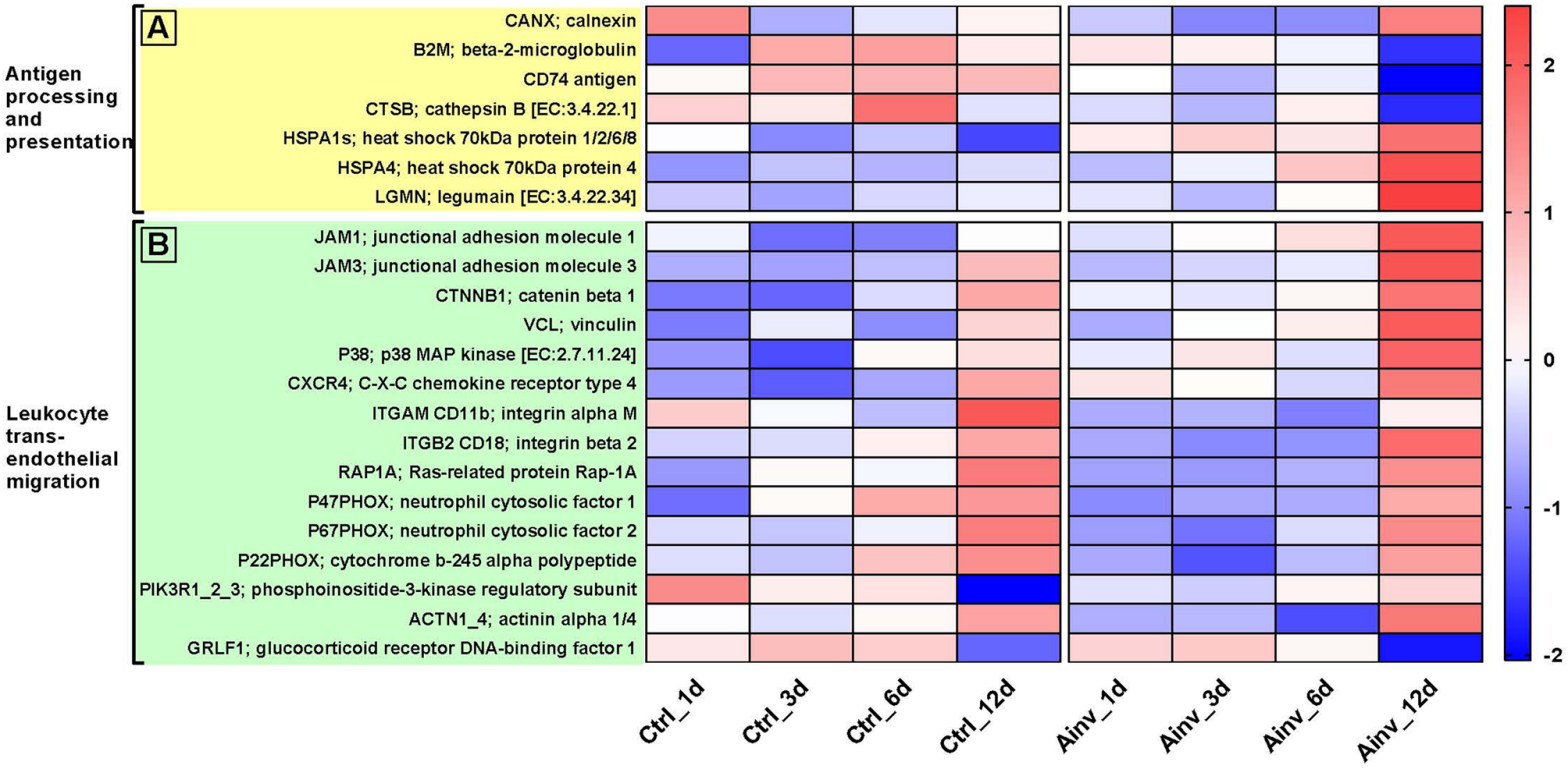
Heatmaps of differentially expressed genes between *Aphanomyces invadans*-infected (Ainv) and control (Ctrl) rohu at 1, 3, 6 and 12 days post-infection, mapping to; (**A**) Antigen processing and presentation and (**B**) Leukocyte transendothelial migration pathways. Each cell in the heatmap represents the average expression level from three independent biological replicates. Colour legend is on a log10 scale. Trinity contig names matching each gene can be found in Suppl. Table 6.

The majority of genes (*CANX*, *B2M*, *CD74* and *CTSB*) in Antigen processing and presentation pathway were downregulated during infection. In contrast, two genes belonging to *HSP70* family including *HSPA1s* and *HSPA4* as well as *LGMN* were constantly upregulated during infection with *A. invadans* (Fig. 4A). Similarly, many genes of Leukocyte transendothelial migration pathway were differentially expressed in Ainv group (Fig. 3B), which included upregulation of the *JAM-1*, *JAM-3*, *CTNNB1*, *VCL*, *P38* and *CXCR4*, whereas, integrins *CD11b* and *CD18*, *RAP1A*, *P47PHOX*, *P67PHOX*, *P22PHOX* and *GRLF1* were downregulated. In addition, *PIK3R1_2_3* as well as *ACTN1_4* were downregulated up to mid stages of infection (Fig. 4B).

**Figure 5:**
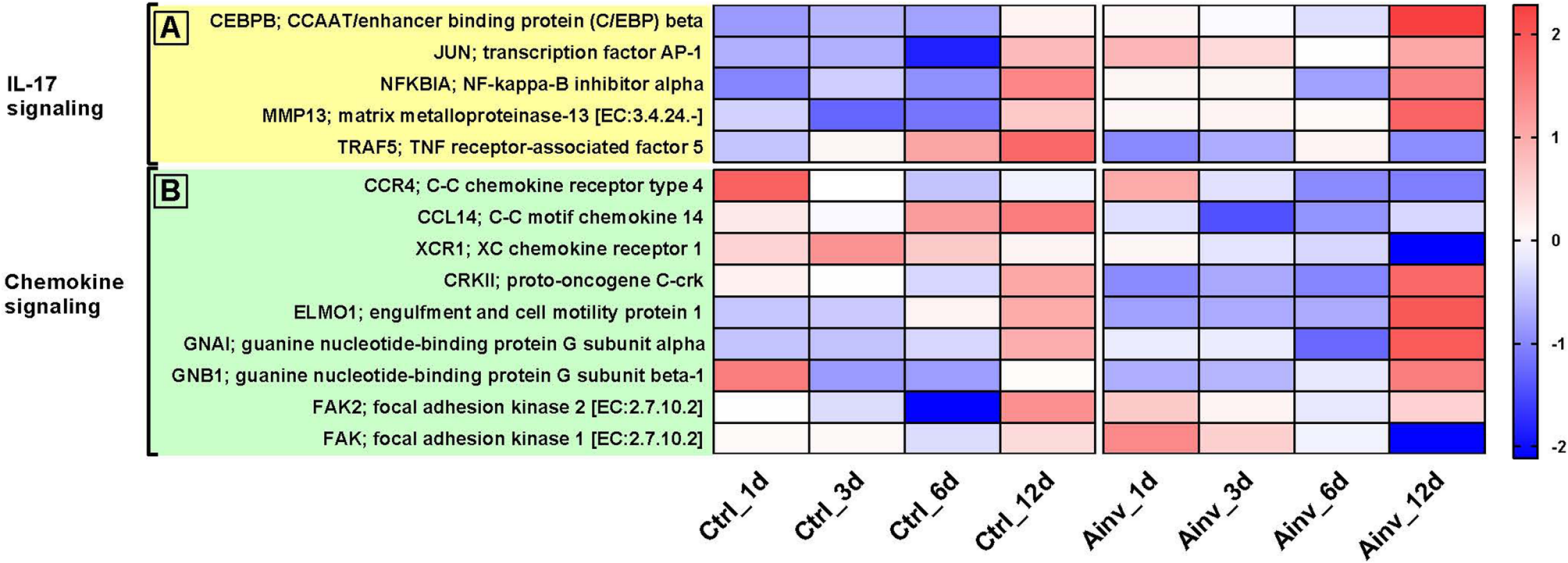
Heatmaps of differentially expressed genes between *Aphanomyces invadans*-infected (Ainv) and control (Ctrl) rohu at 1, 3, 6, 12 days post-infection, mapping to; (**A**) IL-17 signaling and (**B**) Chemokine signaling pathways. Each cell in the heatmap represents the average expression level from three independent biological replicates. Colour legend is on a log10 scale. Trinity contig names matching each gene can be found in Suppl. Table 6.

In the IL-17 signaling pathway, transcription factors *CEBPB*, *JUN*, *NFKBIA* as well as *MMP13* were upregulated, whereas adaptor protein *TRAF5* was found to be downregulated (Fig. 5A). Furthermore, many chemokine receptors like *CCR4*, *CCL14*, *XCR1* along with the adaptor molecules *CRKII* and *ELMO1* were downregulated during initial and mid stages of infection, and in contrast, some G protein signaling molecules such as *GNAI*, *GNB1*, *FAK* and *FAK2* were upregulated in infected fish (Fig. 5B).

**Figure 6:**
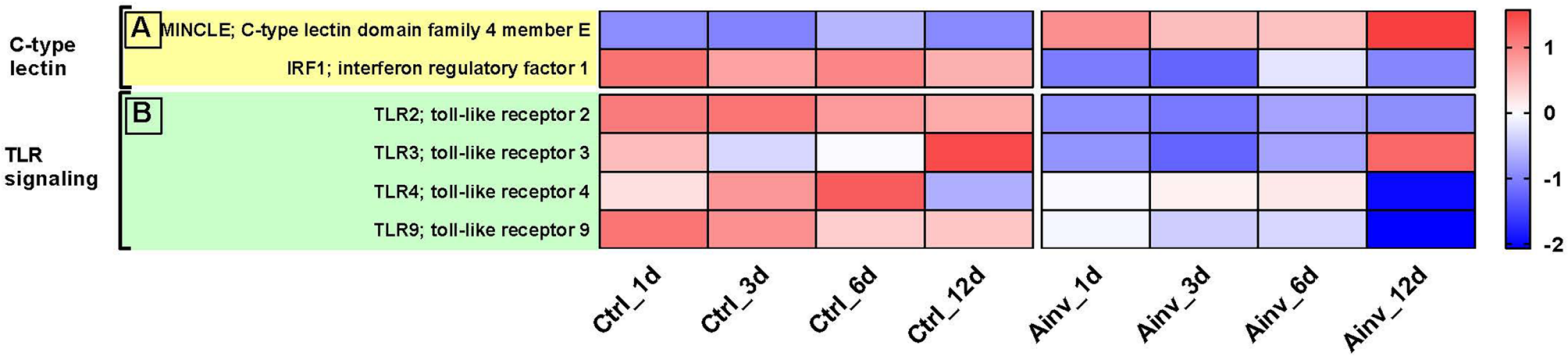
Heatmaps of differentially expressed genes between *Aphanomyces invadans*-infected (Ainv) and control (Ctrl) rohu at 1, 3, 6, 12 days post-infection, mapping to; (**A**) C-type lectin and (**B**) TLR signaling pathways. Each cell in the heatmap represents the average expression level from three independent biological replicates. Colour legend is on a log10 scale. Trinity contig names matching each gene can be found in Suppl. Table 6.

In the C-type lectin receptor signaling pathway, *MINCLE*, a type II transmembrane receptor was found to be upregulated, whereas *IRF1*, a master transcriptional activator of immune responses was found to be downregulated in infected fish (Fig. 6A). Importantly, in fish infected with *A. invadans*, TLRs (*TLR2*, *TLR3*, *TLR4* and *TLR9*) were downregulated at all time points compared to the control group (Fig. 6B).

### Validation of the transcriptome results using qRT-PCR

**Figure 7:**
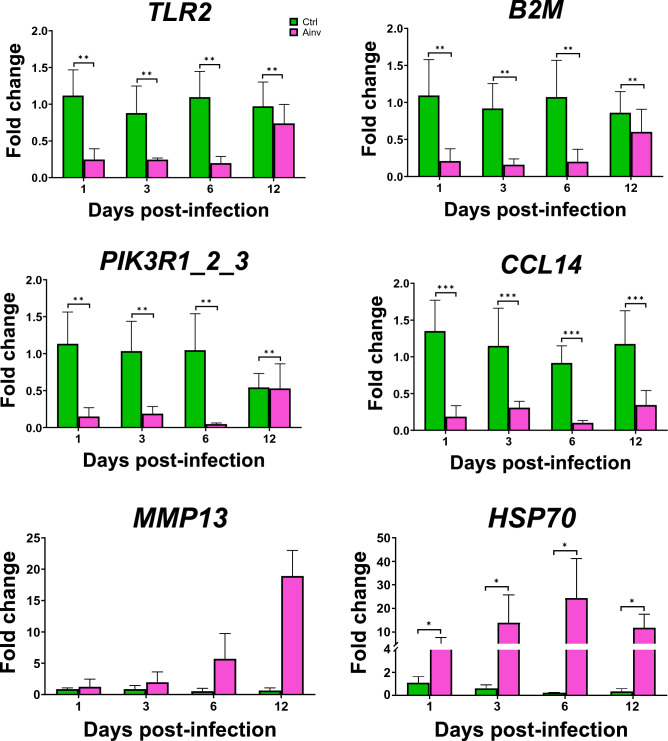
Validation of the selected differentially expressed genes in *Labeo rohita* following *Aphanomyces invadans* infection using qPCR. Gene expression levels were normalized to that of β-actin, and are presented as relative fold change compare with respective control group at 1, 3, 6 and 12 day post-infection. The asterisks (*, *p* < 0.05; **, *p* < 0.01; ***, *p* < 0.001) indicate statistically significant difference when compared with control.

To confirm the results of transcriptome analysis, six DEGs were randomly selected for the qRT-PCR validation test. Among them, *TLR2*, *B2M*, *PIK3R1_2_3* and *CCL14* genes were downregulated in Ainv group compared to the Ctrl. On the other hand, *HSP70* and *MMP13* genes were found to be upregulated in Ainv group in qRT-PCR (Fig. 7).

## Discussion

To date, RNA sequencing has been widely employed to study host transcriptional responses to a number of pathogens^15,21,22^, however, the knowledge regarding the response of the host against *A. invadans* infection is very limited^13^. Therefore, in the present study, the transcriptional response in head kidney of both infected and non-infected rohu was compared at different time points to unravel the dynamic changes in gene expression during the course of infection. Following experimental infection of rohu with *A. invadans* zoospores, a progressive increase in the severity of the gross lesions indicates the successful reproduction of the disease, in line with previous reports^23,24^. For the analysis, de novo transcriptome assembly from RNA-seq data from head kidney of control and *A. invadans*-infected rohu was generated. Previously, in rohu, transcriptome analysis has been carried out for identification of reproduction-related genes^25^.

The time course analysis of RNA-Seq data from head kidney samples of control and experimentally infected rohu revealed 5608 DEGs, which were grouped in the 9 clusters on the basis of expression values. The GO enrichment analysis of cluster 5 and 8 indicated downregulation of unigenes, most of which were involved in immune response. Importantly, maximum downregulation of the unigenes in these clusters was observed at 12 dpi and coincided with severe gross lesions and notably, massive proliferation of oomycete hyphae along with extensive myonecrosis have been reported at later stages of infection^24^. Hence, it can be assumed that the effectors released by proliferating hyphae of *A. invadans* would have been responsible for the observed downregulation of the unigenes. On the other hand, DEGs in clusters 3 and 6 were upregulated with maximum expression at later stages of infection. GO enrichment of these clusters revealed that some of the GO terms especially in cluster 6 were involved in immune response. The overrepresentation of these GO terms appears to be an attempt by host to prevent the invasive spread of the oomycete hyphae, which is however ineffective, in light of the gross lesions that can be observed in infected fish. Similar to our findings, previously Yadav et al.^23^ reported that host responses are not adequate to prevent the invasive spread of *A. invadans* hyphae. In addition, KEGG analysis of DEGs indicated that 390 unigenes were categorised in 21 immune pathways. Some of the important pathways mediating rohu response to *A. invadans* are discussed below.

### Antigen processing and presentation

It is an important immunological process in which MHC class I molecules (MHC I) present foreign peptides derived from degradation of intracellular pathogens, whereas MHC class II molecules (MHC II) are involved in presentation of extracellular antigens^26^, for recognition by T cells^27^. In addition, MHC I molecules can also recognize and present exogenous antigens through a process known as cross-presentation^28^.

In the infected rohu, *CANX* and *B2M* belonging to MHC I were found to be downregulated. As MHC I is generally activated in response to intracellular antigens and is the major route of presentation of viral antigens, downregulation of the above genes suggests a potential intracellular mode of pathogenicity for *A. invadans*. Similar to our findings, modulation of MHC I genes in response to pathogens other than viruses has been reported^29,30^. In addition, *CD74* (I chain) and *CTSB* genes of MHC II were also downregulated. Previously, MHC I and II genes were found to be downregulated in Atlantic salmon following infection with *Saprolegnia parasitica*^31^. Further, suppression of host MHC II molecules has been reported in rainbow trout monocyte/macrophage cell line following infection with oomycete pathogens *Achlya* and *Saprolegnia*^32^. It is important to mention that oomycetes secrete effector proteins that translocate into host cells^33^ and assist the invasion and propagation of the pathogen by reducing the host resistance and overcoming immune responses to the advantage of the pathogens^34,35^. Therefore, it can be inferred that downregulation of the genes belonging to MHC Class I and II in the present study could be a part of immune evasion strategy of the oomycete *A. invadans*. In addition to the downregulation of above genes in this study, a few genes i.e. *HSP70* and *LGMN* were upregulated. HSP70 has been reported to bind antigenic peptides generated within the cell and transports them to the MHC class I molecules present on the cell surface, for presentation to lymphocytes^36^. Besides, *LGMN* is involved in antigen processing for class II MHC presentation^37^. The upregulation of both *HSP70* and *LGMN* suggests that these would be contributing to anti-*A. invadans* response in rohu.

### Leukocyte transendothelial migration

Leukocyte transendothelial migration is a feature of the innate immune system that allows the leukocytes to mobilize into infected tissue to combat the invading pathogen, and is considered as one of the most important signaling pathways in the process of organism-specific immune response^38,39^. In this study, in the infected rohu, the downregulation of leukocyte integrins, namely α (*CD11b*) and β2 (*CD18*) integrins would have prevented their heterophilic interaction of JAMs expressed by endothelial and epithelial cells. In addition to the two integrins, the downregulation of *RAP1A*, *GRLF1*, *ACTN1_4* and *PIK3R1_2_3* would have contributed to the inhibition of leukocyte migration in infected rohu. Besides, *P22PHOX*, *P67PHOX* and *P47PHOX*, which are components of the leukocyte NADPH oxidase complex, were found to be downregulated in infected rohu. These genes are reported to play a role in the production of reactive oxygen species which in addition to their microbicidal activity, also facilitate the migration and adhesion of cells^40,41^. Importantly, *P47PHOX* deficiency in humans is associated with neutrophil dysfunction and chronic granulomatous disease^42^. Therefore, in the present study, it can be assumed that the downregulation of the *P47PHOX* in infected rohu would have contributed to the chronic granulomatous reaction caused by the oomycete. Besides, upregulation of *JAM-A*, *JAM-C*, *CTNNB1* and *VCL* likely contributed to high vascular endothelium cadherin levels^43,44^. On the other hand, the upregulation of *CXCR4* and *P38* would be a part of host defence against *A. invadans*. Previously, *P38* is reported to be activated following cellular stresses and in response to inflammatory cytokines^45^, whereas *CXCR4* plays an important role in recruitment of mesenchymal stem cells and promoting neo-vascularization in wounds^46^. Hence, it can be inferred that modulation of most of above genes would be contributing to susceptibility of rohu to infection with *A. invadans* by inhibiting leukocyte migration to the affected site.

### IL-17 signaling pathway

Interleukin (IL)-17 is a pro-inflammatory cytokine produced mainly by Th17 cells, which promotes immunity to extracellular microbes, especially fungi^47^. In rohu following experimental infection with *A. invadans*, a number of transcription factors involved in mediation of IL-17 downstream signaling including *CEBPB*, *JUN* as well as *NFKBIA* were upregulated. In earlier studies, it has been reported that upregulation of *NFKBIA* directly reflects activation of NF-κB signaling^48^. Therefore, it can be assumed that NF-κB transcription factors are upregulated in infected rohu. It is to be noted that a distinctive inflammatory response occurs against the invading oomycete *A. invadans*^49,50^ and importantly, transcription factors play a key role in inflammatory processes^48,51,52^. The upregulation of transcription factors in the present study is crucial for the inflammatory response during *A. invadans* infection. Besides, upregulation of *MMP13* in the infected rohu is in accordance with earlier report of abundant expression of *MMP13* in chronic cutaneous ulcers^53^. Additionally, *MMP13* has been reported to be upregulated in *Ictalurus punctatus* and *Paralichthys olivaceus* following infection with *Edwardsiella ictaluri*^54^ and *E. tarda*^55^, respectively. Jiang et al.^54^ also suggested that catfish *MMP13* helps to elicit innate immunity against the pathogen in the initial stages, whereas in later stages, it stimulates repair of the damaged tissue through tissue remodelling. In contrast, the downregulation of *TRAF5* in *A. invadans* infected rohu would have reversed the inhibitory effect of *TRAF5* on Th17 cell development, as reported earlier^56^. The authors also suggested that Th17 cells play a critical role in immunity against bacterial and fungal pathogens. Therefore, the modulation of genes in IL-17 signaling suggest an attempt of the host for resisting the oomycete infection.

### Chemokine signaling pathway

The chemokine superfamily of proteins serves to coordinate a variety of immune system functions that link both innate and adaptive immunity^57^ and is best known for its key role in the recruitment and retention of leukocyte populations in both homeostasis and immune responses to pathogens^58^. The increased expression of *CCR4* and its ligands has been reported in granulomatous response^59^. Therefore, the downregulation of *CCR4* in the present study would have prevented an effective granulomatous response that could contain the invasive spread of oomycete hyphae. In addition, in infected rohu, downregulation of *CRKII*, *CCL14*, *PKA* and *ELMO1* genes would have inhibited chemotaxis of leukocytes to the site of injury. Furthermore, *XCR1* is reported to play a key role in activation of CD8 + T cells and cross-presentation of exogenous antigens^60,61^. Concomitantly, downregulation of *XCR1* in infected rohu likely inhibits the activation of cytotoxic lymphocytes and cross-presentation of oomycete antigens. On the other hand, upregulation of *GNAI*, *GNB1*, *ADCY3*, *FAK* and *FAK2* is an attempt on the part of host for facilitating migration of leukocytes to the site of injury.

### C-type lectin receptor signaling pathway

C-type lectin receptor (CLR)-induced intracellular signal cascades are indispensable for the initiation and regulation of anti-fungal immunity^62^. *MINCLE*, is expressed predominantly on macrophages, and is reported to potentiate pro-inflammatory cytokine production^63^. Previously, mice lacking *MINCLE* showed a significantly increased susceptibility to systemic candidiasis^63^. Therefore, it can be inferred that although upregulation of *MINCLE* in infected rohu plays an important role against *A. invadans* infection, it is not sufficient to control the infection. Besides, in teleosts, *IRF1* can bind directly to type I IFN promoter and activate type I IFN-mediated antiviral response^64,65^. The downregulation of *IRF1* along with downregulation of some of genes of MHC-1, as discussed earlier, would have hampered the response to oomycete antigens and therefore, *A. invadans*-infected fish could be prone to infection with viruses, as reported previously^66^.

### Toll-like receptor (TLRs) signaling pathway

TLRs are a major class of pattern-recognition receptors that recognise pathogen-associated molecular patterns (PAMPs), resulting in the production of inflammatory cytokines. These cytokines help in modulating innate and adaptive immunity as well as inflammatory responses^67^. In the current study, following infection of rohu with *A. invadans*, *TLR2*, *TLR3*, *TLR4* and *TLR9* were downregulated. Previously, TLRs particularly *TLR2* and *TLR4* have been reported to play an important role in recognizing ligands from different fungal pathogens^68,69^. Moreover, it has been reported that *Aspergillus fumigatus* is able to evade immune recognition during germination through loss of TLR4-mediated signaling^70^, and absence of TLR4-mediated signals has been reported to result in increased susceptibility to disseminated candidiasis in TLR4-defective mice^71^. Furthermore, lack of *TLR2* has also been reported to impair the early recruitment as well as antimicrobial capacity of neutrophils against *A. fumigatus*^67,68^. In addition, germinated zoospores of *A. invadans* have been reported to proliferate massively in fingerlings of Indian major carp and induce extensive necrotizing myositis without the usual infiltration of inflammatory cells around hyphae^72^. Therefore, from the present study, it can be assumed that *A. invadans* was able to evade immune recognition by the host through downregulation of *TLR2* and *TLR4*. Interestingly, in contrast, upregulation of *TLR2* and *TLR4* has been reported in rainbow trout following infection with *S. parasitica*^73^. The observed variation in expression pattern of TLRs could be due to differences between oomycete and host species as well as routes of infection^74,75^. Additionally, *TLR3* and *TLR9* are intracellular receptors which recognize nucleic acids mainly from viruses and also from bacteria^76–78^. *TLR9* has been reported to regulate inflammation following infection with *Aspergillus*^79^. The observed downregulation of both the TLRs could be due to effectors produced by *A. invadans*.

The RNA-Seq results of selected DEGs in infected rohu were further validated by qRT-PCR, confirming that RNA-Seq data of the study are accurate and therefore, can be used for inferring biological relevance.

## Conclusions

Time course analysis of RNA-Seq data generated from head kidney of rohu challenged with *A. invadans* revealed 5608 differentially expressed genes, of which, 390 genes were involved in 21 immune-related pathways. Our findings provide information on the dynamics of host–pathogen interaction, and changes in expression pattern of genes along with progression of infection. In particular, this analysis helped us in identifying immune genes that showed consistent downregulation with time in infected animals (i.e. clusters 5 and 8), and were temporally concomitant with the appearance of severe gross lesions. In general, most of the differentially expressed immune genes were related to Antigen processing and presentation, Leukocyte transendothelial migration, Chemokine signaling, IL-17 signaling, C-type lectin receptor signaling and Toll-like receptor signaling pathways. The upregulation of some immune-related genes in the affected pathways indicates an attempt by rohu to resist *A. invadans* infection, however, these responses might not be effective enough to prevent the invasive growth of oomycete hyphae. On the other hand, the downregulation of the majority of immune genes in the affected pathways suggested that *A. invadans* was able to modulate the host immune response to its advantage and thereby, successfully establish an infection. The results of the present study provide insights in our understanding of molecular mechanisms of the susceptibility of rohu to infection with *A. invadans* and would serve as molecular resources for future research related to immunoprophylaxis.

## Materials and methods

### Experimental animals and oomycete pathogen

Rohu juveniles (36.8 ± 2.0 g and 14.99 ± 2.0 cm, n = 24) were obtained from Fish Germplasm Resource Centre of ICAR-National Bureau of Fish Genetic Resources (ICAR-NBFGR), Lucknow. The fish were randomly distributed in two independent fibre-reinforced plastic (FRP) tanks (12 fish per tank) filled with aerated 300 l freshwater at 28 °C. Tanks were supplied by a static water system with approximately 30% daily water exchange. Fish were acclimatized for one week prior to experimental infection and fed twice a day with a commercial diet (ABIS Exports India Pvt. Ltd., Chhattisgarh, India) at 2% body weight per day. Fish were held under natural photoperiod according to the winter season at 16.0 ± 2.1 °C, 5.54 ± 0.68 mg l^-1^ dissolved oxygen and pH 7.8 ± 0.43.

The *A. invadans* strain INM20101, isolated previously from *Cirrhinus mrigala* during an EUS outbreak^22^ and maintained at ICAR-NBFGR, was used for challenge. A suspension of motile secondary zoospores was prepared following Lilley et al.^11^ and the concentration was adjusted to 10^4^ spores ml^-1^ in autoclaved pond water (APW).

### Experimental infection

After acclimatization, fish (n = 12) were injected intramuscularly (flank region below the anterior part of the dorsal fin) with 100 µL of APW containing 1000 zoospores of *A. invadans*. Similarly, the control fish (n = 12) were injected with the same volume of sterile APW. Following injection, 6 fish (3 control and 3 *A. invadans*-infected fish) were randomly selected and sampled on 1, 3, 6 and 12 days post infection (dpi). At each time point, following euthanasia with MS-222 (Sigma-Aldrich, St. Louis, MO, USA), the head kidney was dissected from control and *A. invadans*-infected fish, snap frozen in liquid nitrogen and subsequently transferred to − 80 °C.

### Total RNA extraction, cDNA library construction, and sequencing

Total RNA was extracted from head kidney tissue (n = 24, three biological samples per treatment per time point), using RNeasy kit (Qiagen, Valencia, CA, USA). The samples were designated as *A. invadans*-infected, 'Ainv', and control, 'Ctrl' rohu at 1 dpi ('Ainv 1d', 'Ctrl 1d'), 3 dpi ('Ainv 3d', 'Ctrl 3d'), 6 dpi ('Ainv 6d', 'Ctrl 6d') and 12 dpi ('Ainv 12d', 'Ctrl 12d'). The extracted RNA was treated with RNase-free DNase I (Thermo Fisher Scientific, Logan, Utah, USA), and its quality and integrity verified using a DeNovix DS-11 Spectrophotometer (DeNovix Inc., Wilmington, Delaware, USA) and Agilent 2100 Bioanalyzer system (Agilent Technologies, CA, USA), respectively. RNA samples having RIN value > 7 were sent to M/s SciGenom Pvt. Ltd. (Kochi, India), where a paired end sequencing 2 × 100 bp was carried out on a Illumina Hiseq 2500 platform (Illumina Inc., San Diego, CA, USA).

### Transcriptome assembly and annotation

Raw reads obtained from all the libraries were quality checked using FastQC/v0.11.3 (https://www.bioinformatics.babraham.ac.uk/projects/fastqc/), and low-quality reads were removed using Trimmomatic/ v0.38 with default parameters^80^. The remaining reads were used for de novo transcriptome assembly using Trinity/v2.8.4 with default parameters^81^. The redundancy of the generated assembly was reduced using CD-HIT/v4.6.4^82^ and the quality of the generated assembly was assessed using gVolante (https://gvolante.riken.jp/analysis.html) against core vertebrate genes database using a BUSCO orthology pipeline^83,84^. For expression count of each contig, reads from control and infected samples at each time point were mapped to the generated assembly using Kallisto/v0.43.1^85^. The count matrix was imported into R/v3.5.067 and filtered to keep contigs only with at least five reads in 12 or more samples; subsequently the functions 'betweenLaneNormalization' and 'RUVs' (with k = 9) from the R package RUVSeq/v1.14.0 were used to normalize the dataset and remove unwanted variation^86^. After the normalization, an additional filtering step was performed to keep only contigs with at least one normalised count in 12 or more samples, to avoid having many contigs with 0 count that would result in convergence problem with the subsequent time course analysis. After filtering, Principal Component Analysis (PCA) was performed in R using the package RUVSeq.

The filtered transcriptome was annotated against NCBI UniProt database with BlastX/v2.8.0 (e-value 1E-05 and default options) and InterPro Scan/v5.29–68.0 (default options)^87,88^. Hits were imported in OmicsBox/v1.2 (formerly Blast2GO, https://www.biobam.com) followed by mapping (GO version Jan 2020), annotation and InterProScan analysis in parallel with default options. The annotation file was generated by merging the annotated BLAST results with InterProScan results. Raw sequence data associated with this project have been deposited at NCBI (bioproject accession number PRJNA612592 and SRA accession numbers SRR11306747-SRR11306770).

### Time course expression analysis

The RUVSeq normalized count matrix and annotated transcriptome assembly of rohu were imported in OmicsBox and Time Course (TC) differential expression analysis was performed with default parameters. Statistical significance was identified at *p* < 0.05 with an R^2^-cutoff > 0.7. All clusters containing genes differentially regulated in *A. invadans-*infected group were merged in OmicsBox to perform enrichment analysis with Fisher’s Exact test at FDR *p* < 0.05. All significant features from TC analysis were submitted to the KEGG Automatic Annotation Server (KAAS, https://www.genome.jp/tools/kaas/) to retrieve KEGG pathway maps for each contig using single-directional best hit (SBH) method^20^. The expression level for each gene in each pathway was extracted from OmicsBox and used to plot the heatmaps in Prism/v8.0.2 (GraphPad Software, California USA, www.graphpad.com) after log_10_ transformation of the data.

### Validation of bioinformatic analysis through qRT-PCR

The expression of selected differentially expressed genes (*TLR2*, *B2M*, *PIK3R1_2_3*, *CCL14*, *MMP13*, *HSP70*) was further confirmed by qRT-PCR. The RNA, extracted previously, was used for cDNA synthesis using RevertAid H minus First-Strand cDNA Synthesis Kit (Thermo Scientific, USA). qRT-PCR was carried out on a StepOne Plus Real-Time PCR system (Applied Biosystems, Foster City, CA, USA). Briefly, 20 μl reaction mixture consisted of 10 μl 2 × SYBR Green Master mix (Applied Biosystems), 0.5 μl of each gene-specific primer (5 pmol), 4 μl of nuclease-free water and 5 μL of (1:10) diluted cDNA as a template. Gene-specific primers (Suppl. Table 7) were designed using Primer 3.0 software (Applied Biosystems). The qRT-PCR cycling conditions included a holding stage of 95 °C for 2 min followed by 40 cycles of denaturation at 95 °C for 30 s and annealing/extension at 60 °C for 30 s. Melt-curve analysis of each primer was performed following PCR amplification, to rule out the possibility of primer dimers and non-specific product formation. Each reaction was run in duplicate (technical replicates) along with NTC (no template control). β-actin was used as reference gene for data normalization. The comparative 2^−ΔΔCt^ method based on C_T_ values was used to determine the expression level of the each gene^89^.

### Statement

All experiments and methods were performed in accordance with relevant guidelines and regulations. The animal care and experimental challenge were approved by the Institutional Animal Ethics Committee of ICAR-National Bureau of Fish Genetic Resources, Lucknow (registration number 909/GO/Re/S/05/CPCSEA).

## Supplementary information

Supplementary Information

Supplementary Information

Supplementary Information

Supplementary Information

Supplementary Information

Supplementary Information

Supplementary Information

Supplementary Information

Supplementary Information

Supplementary Information

## Data Availability

Raw sequence data associated with this project have been deposited at NCBI with bioproject accession number PRJNA612592 and SRA accession numbers SRR11306747-SRR11306770.
