# Supplementary material for: Molecular insights into the mechanisms of susceptibility of Labeo rohita against oomycete Aphanomyces invadans
Source: Sci Rep. 2020 Nov 11;10:19531. doi: 10.1038/s41598-020-76278-w (PMC7658212; doi:10.1038/s41598-020-76278-w)
Supplement: Supplementary file 1 — Supplementary Information [file 41598_2020_76278_MOESM1_ESM.docx]

**Molecular insights into the mechanisms of susceptibility of *Labeo rohita* against oomycete *Aphanomyces invadans***

P.K. Pradhan^1#^, Dev Kumar Verma^1#^, Luca Peruzza^2,6#^, Shubham Gupta^1^, Syed Assim Haq^1^, Sergei V. Shubin^3^, Kenton L. Morgan^4^, Franziska Trusch^5,7^, Vindhya Mohindra^1^, Chris Hauton^2^, Pieter van West^5^, Neeraj Sood^1^*

^1^ICAR-National Bureau of Fish Genetic Resources, Canal Ring Road, P.O. Dilkusha, Lucknow-226 002, Uttar Pradesh, India

^2^School of Ocean and Earth Science, University of Southampton, Waterfront Campus, European Way, Southampton, SO14 3ZH, United Kingdom

^3^College of Science, Swansea University, Singleton Park, Swansea SA2 8PP, United Kingdom

^4^The Institute of Veterinary Science, University of Liverpool, Leahurst Campus, Neston, CH64 7TE, Liverpool, United Kingdom

.

^5^International Centre for Aquaculture Research and Development, Institute of Medical Sciences, University of Aberdeen,Foresterhill, Aberdeen AB25 2ZD, Scotland, United Kingdom.

^6^Present adress: Department of Comparative Biomedicine and Food Science, University of Padova, Viale dell’Università 16, 35020, Legnaro (PD) , Italy

^7^Present address: University of Dundee, School of Life Sciences, Department of Plant Sciences (@ James Hutton Institute), Invergowrie, Dundee DD2 5DA, Scotland, United Kingdom

**Supplementary Material**

**Supplementary Fig. 1.** Principal Component Analysis showing the differences between the independent biological samples analyzed from *A. invadans*-infected 'Ainv' and control 'Ctrl' *Labeo rohita* at different time points (i.e. 1, 3, 6 and 12 days post-infection). Each symbol represents an independent biological replicate sample from different treatments at different time points.

**Supplementary Fig. 2.** Distribution of differentially expressed genes (DEGs) of *Labeo rohita* head kidney transcriptome categorised by the Kyoto Encyclopedia of Genes and Genomes (KEGG) database.
