# Supplementary figures and images for: Molecular insights into the mechanisms of susceptibility of Labeo rohita against oomycete Aphanomyces invadans
Source: Sci Rep. 2020 Nov 11;10:19531. doi: 10.1038/s41598-020-76278-w (PMC7658212; doi:10.1038/s41598-020-76278-w)

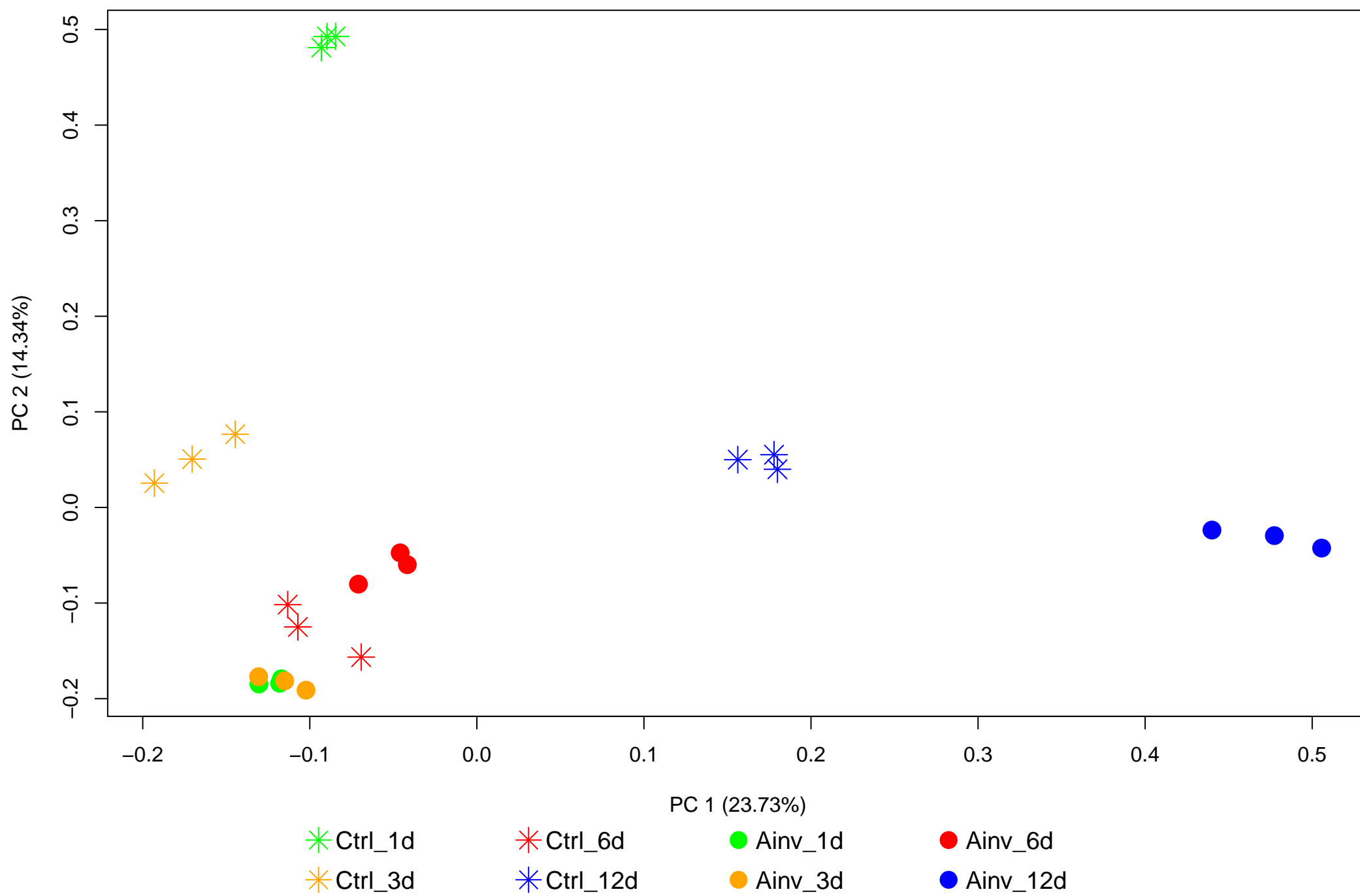

Supplement: Supplementary file 2 — Supplementary Information [file 41598_2020_76278_MOESM2_ESM.pdf]
