# Supplementary material for: Molecular insights into the mechanisms of susceptibility of Labeo rohita against oomycete Aphanomyces invadans
Source: Sci Rep. 2020 Nov 11;10:19531. doi: 10.1038/s41598-020-76278-w (PMC7658212; doi:10.1038/s41598-020-76278-w)
Supplement: Supplementary file 3 — Supplementary Information [file 41598_2020_76278_MOESM3_ESM.pptx]

## Slide 1
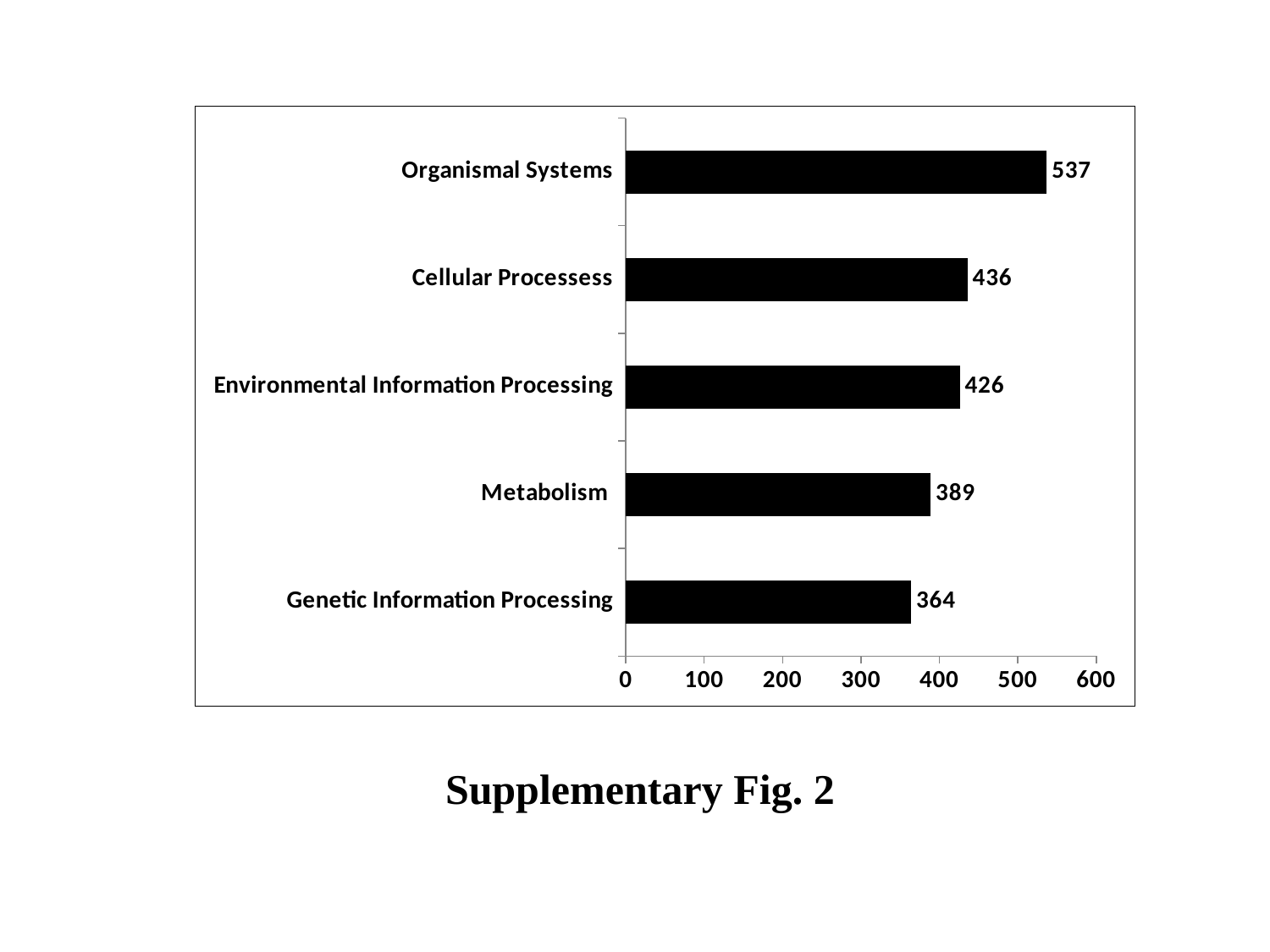

### Chart
| Category | |
|---|---|
| Genetic Information Processing | 364.0 |
| Metabolism | 389.0 |
| Environmental Information Processing | 426.0 |
| Cellular Processess | 436.0 |
| Organismal Systems | 537.0 |Supplementary Fig. 2
